# Anthropometric measurements as predictors of nutritional status in black South African women during pregnancy

**DOI:** 10.1111/jog.16184

**Published:** 2024-12-25

**Authors:** Christen R. Erasmus, Niren R. Maharaj, Anil A. Chuturgoon

**Affiliations:** ^1^ Department of Medical Biochemistry University of KwaZulu‐Natal Durban South Africa; ^2^ Department of Obstetrics and Gynaecology University of the Free State South Africa

**Keywords:** anthropometry, nutritional status, obesity, overweight

## Abstract

**Background:**

Nutritional risk assessment is an essential component of primary health care screening, especially for pregnant women. The aim of this study was to investigate the relationship between maternal body mass index (BMI) and maternal anthropometric measurements in black South African pregnant women, both with and without human immunodeficiency virus (HIV).

**Materials and Methods:**

A cross‐sectional observational study design was used. Two hundred black South African pregnant women were recruited of which 90 were HIV‐infected and 110 were HIV‐uninfected. The anthropometric measurements assessed included mid‐upper arm circumference (MUAC), tricep skinfold (TSF), subscapular skinfold (SSF), mid‐arm muscle circumference (MAMC), wrist circumference (WC), frame size, and BMI.

**Results:**

Maternal age was significantly associated with changes in maternal anthropometric measurements. Maternal BMI was significantly correlated with other maternal anthropometric measurements including MUAC, TSF, SSF, MAMC, WC, and frame size. The anthropometric measurements that were found to be accurate for assessing obesity in pregnancy included TSF (≥20.75 mm), SSF (≥21.75 mm), MAMC (≥25.23 cm), and WC (≥16.25 cm). Additionally, SSF (≥15.75 mm) and MAMC (≥23.35 cm) could be used to assess for overweight nutritional status. Lastly, frame size could be used to assess for underweight (≥10.05) and normal (≥9.95) nutritional status. No significant anthropometric differences were observed between the HIV‐infected pregnant women and the HIV‐uninfected pregnant women in this study.

**Conclusion:**

Surrogate anthropometric measurements offer a simple solution for assessing nutritional status in pregnant women. MUAC was the most accurate method for identifying overweight and obesity. Furthermore, maternal HIV status did not affect the anthropometric measurements.

## BACKGROUND

Nutritional risk assessment is an essential component of primary health care screening systems, especially for the identification of nutritionally at‐risk pregnant women.^1^ Nutritional status during pregnancy can be classified by using the pregnant women's anthropometric measurements.^2^ Overweight and obesity during pregnancy have become major public health concerns in low‐ to middle‐income countries, including South Africa.^3^ This is particularly important in light of the consistently high prevalence of HIV among pregnant women in recent years, with an estimated national prevalence rate of 29.1% in 2019.^4^

Overweight and obesity during pregnancy are most frequently assessed using the body mass index (BMI) classification, with a score of ≥25 and ≥30 kg/m^2^, respectively.^5^, ^6^, ^7^, ^8^ However, in resource‐limited settings such as in South Africa, surrogate measurement methods are needed to easily assess nutritional status when assessors are unable to measure BMI. Hence, other anthropometric studies have been conducted in pregnant women using other body measurements, such as arm circumferences and skinfold thickness measurements.^9^, ^10^, ^11^, ^12^, ^13^, ^14^ However, there is a need to further validate the use of these surrogate measurement methods to accurately assess and predict nutritional status in pregnancy, especially among pregnant black South African women living with and without HIV. Currently, there are no South African based studies that have investigated the correlation between maternal BMI and maternal mid‐arm muscle area or maternal skinfold measurements and their accuracy as predictors of nutritional status in black South African pregnant women. Therefore, this study sought to provide insight into whether there is an association between maternal BMI and other maternal anthropometric measurements among pregnant black South African women; and to identify measurement cut‐offs that accurately predict each nutritional status group.

HIV and antiretroviral treatment (ART) in pregnancy are associated with an increased risk for cardio‐metabolic abnormalities and changes in body weight.^3^ Pregnancy is an anabolic process where there is physiological alterations of the delivery, metabolism, and storage of nutrients to meet the needs of the growing feotus and mother.^15^ In combination with HIV, these metabolic processes are further altered which may cause shifts in fat mass and fat‐free mass.^16^, ^17^ Hence, HIV and ART have been associated with body composition changes in pregnant women such as wasting and weight gain.^12^, ^18^, ^19^, ^20^, ^21^ Therefore, since HIV and ART in pregnancy are also associated with an increased risk for changes in body composition, this study also investigated the anthropometric differences between pregnant women living with and without HIV.

## METHODS

### Sample selection and study population

A cross‐sectional observational retrospective study design was employed. Sample selection was conducted at Prince Mshiyeni Memorial Regional Hospital (PMMH), which is situated in Umlazi within the eThekweni municipality, KwaZulu‐Natal, South Africa. The catchment area for the hospital includes both rural and urban geographical areas. Pregnant women admitted to the labor ward were approached to participate in this study from April 2019 to December 2019. The inclusion criteria for this study were as follows: (1) ≥18 years of age; (2) pregnant females; (3) black South African citizen; (4) clinically stable; (5) able to stand without assistance; and (6) provided verbal and written consent to participate in the study. A total of 458 pregnant women participated in the study, but 245 subjects met the inclusion criteria and of these 45 declined to participate. Hence, a total of 200 subjects met all the inclusion criteria, where 90 were HIV‐infected and 110 were HIV‐uninfected. The HIV‐infected pregnant women were compliant with the prescribed fixed‐dose combination (FDC) ART. The prescribed treatment was in accordance with the South African prevention of mother‐to‐child transmission (PMTCT) protocol and the South African ART guidelines.^22^ The HIV‐infected pregnant women received tenofovir (TDF) + emtricitabine (FTC) or lamivudine (3TC) + efavirenz (EFV) as FDC.^22^, ^23^ Most of the women (98.0%) had a singleton pregnancy, while 2.0% had twin pregnancies.

### Maternal anthropometric assessment

Anthropometric measurements were conducted by a level 1 dietician who was International Society for the Advancement of Kinanthropometry (ISAK) trained. To avoid anthropometric measurement errors, all measurements were conducted by the same researcher, taken three times, and recorded to the nearest 0.1 cm/mm/kg.^24^, ^25^ The mean of the two closest values was recorded. All measurements were conducted on the right side of the body unless indicated.^24^, ^25^

#### Body mass index

The standing height (SH) measurement was measured via stretch stature methodology using a calibrated portable stadiometer (Seca) with a sliding headboard.^26^ The weight measurement was taken using the actual body weight (ABW) methodology using a portable calibrated scale (Seca, with a maximum weight threshold of 250 kg).^27^ The scale was calibrated before the commencement of the study. BMI was calculated using the weight of the mother post‐delivery. The BMI was then interpreted, and the pregnant women were categorized according to the following classifications: (1) underweight, BMI of <18.5 kg/m^2^; (2) normal, BMI of between 18.5 and 24.9 kg/m^2^; (3) overweight, BMI of between 25.0 and 29.9 kg/m^2^; and (3) obese, BMI of ≥30.0 kg/m^2^.^28^

#### Mid upper arm circumference

The MUAC measurement was assessed using a Seca measuring tape. The MUAC was measured on the left and right arm and was determined by measuring the linear distance between the acromial and radial sites, with the arm muscle relaxed and the arm extended to their side. The midpoint between these two sites was called the mid‐acromial‐radiale. The circumference of the arm was measured at the level of the mid‐acromial‐radiale, perpendicular to the long axis of the arm. The MUAC is the sum of muscle tissue and subcutaneous fat, and it can be used as an indicator of maternal nutritional status, body composition, and arm thickness.^10^ The MUAC (right) was interpreted according to percentile readings (Table S1, Supporting Information).^29^, ^30^

#### Tricep skinfold thickness and subscapular skinfold thickness

The skinfold thickness measurements were assessed using a caliper. The TSF site is the point on the posterior surface of the right arm, in the mid‐line, at the level of the mid‐acromial‐radiale landmark. The TSF measurement was taken parallel to the long axis of the arm at the TSF site. The SSF site was located by palpating the inferior angle of the scapula with the left thumb in order to find the under most tip. The subject must assume a relaxed standing position with their arms hanging by their sides. The SSF was taken on the right‐hand side with the fold running obliquely downwards at the SSF site. The TSF (right) and SSF (right) measurements were interpreted according to percentile readings to determine the fat stores (Table S1).^29^, ^31^

#### Mid‐arm muscle circumference

The MAMC was used for the muscle‐associated measurement, which was determined by plotting the right TSF and right MUAC measurement on the adult nomogram.^30^ The value obtained was interpreted using percentile readings to determine the muscle stores (Table S1).^31^, ^32^

#### Frame size

The frame size was determined using the frame size calculation (frame size [cm]: [height (cm)/wrist circumference (WC) (cm)]).^33^ The frame size ranges from small to large (Table S2).^34^ The WC was measured among participants in the relaxed standing position. The WC site was determined by measuring the minimal circumference of the right wrist perpendicular to the long axis of the forearm, distal to the styloid processes.^33^

### Statistical analysis

Data were captured using Microsoft Excel and continuous variables were represented as arithmetic mean (x̅) and standard deviation (SD). Categorical data were presented as percentages (%). Data were categorized in relation to HIV status, that is (HIV‐infected or HIV‐uninfected) and nutritional status (underweight, normal, overweight, and obese). Statistical analysis was performed using the statistical software packages, GraphPad Prism 5, and IBM SPSS for Windows version 27. The level of significance (α) used in the statistical analysis was *p* < 0.05. The parameters that were included in the statistical analysis were: (i) BMI; (ii) MUAC (right); (iii) TSF (right); (iv) SSF (right); (v) MAMC (right); (vi) WC (right); and (vii) frame size. The statistical tests included: (i) Fisher's exact test (two categories) and the *χ* ^2^ test (more than two categories) to investigate the comparison between categories; (ii) the Pearson correlation coefficient for data with normal distribution was used to identify the strength of association between variable means; (iii) the Spearman's rank‐order correlation coefficient for data with non‐normal distribution was used to identify the strength of association between variable means (normality was tested using the Kolmogorov–Smirnov test or the Shapiro–Wilk test); (iv) the Mann Whitney *t*‐test was used for comparison between two variable means; (v) one‐way ANOVA was used for the analysis of variance between the variable means; and (vi) area under the curve (AUC) of the receiver operator characteristic (ROC) curves was used to investigate the accuracy of using the anthropometric measurements and defining nutritional status cut‐off points. The interpretation of the AUC was as follows: (i) 0.5 equal to chance; (ii) <0.6 was an inaccurate test of no diagnostic value; (iii) >0.6 and <0.7, interpreted as a poor test; (iv) ≥0.7 and <0.8, interpreted as an acceptable or fair test; (v) ≥0.8 and <0.9, interpreted as an excellent test; and (vi) ≥0.9, interpreted as an outstanding test.^35^ The appropriate cut‐off point for each anthropometric measurement was defined by using Youden's index and calculating the Youden's J statistic (sensitivity + specificity − 1) for each cut‐off measure.^34^ The absolute value of the correlation coefficient was interpreted as follows: (i) ≥0.0 and <0.2, interpreted as a very weak relationship; (ii) ≥0.2 and <0.4, interpreted as a weak relationship; (iii) ≥0.4 and <0.6, interpreted as a moderate relationship; (iv) ≥0.6 and <0.8, interpreted as a strong relationship; and (v) ≥0.8 and ≤1.0, interpreted as a very strong relationship.

### Ethics approval and informed consent

The study was approved by the Biomedical Research Ethics Council (BREC) of the University of KwaZulu‐Natal (UKZN) (BE269/18), KwaZulu‐Natal Department of Health (KZNDOH) (HRKM261/18), and PMMH (29/RESH/2018). All the participants in this study had provided verbal and written consent, participated voluntarily, did not receive incentives, and had the right to withdraw at any stage of the study. All methods were performed in accordance with the guidelines and regulations of the declaration of Helsinki.

## RESULTS

### Correlation of parameters with BMI

The study included 200 black South African women who were pregnant and were categorized according to their nutritional status (Table 1; Table S3). They had a mean gestational age of 37.7 weeks and a mean age of 27.0 years. There was no significant difference (*p* = 0.0778) in the mean maternal age of the participants across all the nutritional status groups. However, there were weak correlations identified between age and all the maternal anthropometric parameters (Table 2). There were strong correlations identified between maternal BMI and MUAC (left) (*r* = 0.9144; 95%CI 0.8883–0.9346; *p* < 0.0001), as well as between maternal BMI and MUAC (right) (*r* = 0.9106; 95%CI 0.8833–0.9317; *p* < 0.0001). There was a strong correlation identified between maternal BMI and TSF (right) measurements (*r* = 0.8065; 95%CI 0.7516–0.8503; *p* < 0.0001) among the pregnant women. There was a strong correlation between maternal BMI and SSF (right) measurements (*r* = 0.7375; 95%CI 0.6663–0.7953; *p* < 0.0001) among the pregnant women. There was a strong correlation between maternal BMI and MAMC (right) measurements (*r* = 0.7964; 95%CI 0.7390–0.8423; *p* < 0.0001) among the pregnant women. There was a strong correlation between maternal BMI and WC (right) measurements (*r* = 0.6822; 95%CI 0.5998–0.7503; *p* < 0.0001) and a strong correlation between maternal BMI and frame size (*r* = −0.6016; 95%CI −0.6837 to −0.5044; *p* < 0.0001) among the pregnant women.

**TABLE 1:** Anthropometric data for pregnant black South African females, categorized according to nutritional status.

Characteristics	All pregnant females	Maternal BMI^a^				
		Underweight	Normal	Overweight	Obese	*p*‐Value
Age	*n* = 200	*n* = 19	*n* = 84	*n* = 52	*n* = 45	
Mean (SD) (years)	27.0 (5.6)	24.9 (5.0)	26.3 (5.6)	27.5 (5.4)	28.3 (5.5)	0.0778
Gestational age	*n* = 200	*n* = 19	*n* = 84	*n* = 52	*n* = 45	
Mean (SD) (weeks)	37.7 (2.8)	35.2 (4.0)	37.7 (2.5)	37.8 (2.6)	38.4 (2.0)	0.0004
MUAC (left)	*n* = 199	*n* = 19	*n* = 83	*n* = 52	*n* = 45	
Mean (SD) (cm)	30.6 (5.1)	24.1 (1.6)	28.0 (2.3)	31.3 (2.4)	37.2 (4.9)	<0.0001
MUAC (right)	*n* = 198	*n* = 19	*n* = 82	*n* = 52	*n* = 45	
Mean (SD) (cm)	30.7 (5.1)	24.4 (1.8)	28.1 (2.4)	31.4 (2.3)	37.4 (4.7)	<0.0001
TSF (right)	*n* = 198	*n* = 19	*n* = 82	*n* = 52	*n* = 45	
Mean (SD) (mm)	19.5 (6.7)	11.1 (2.5)	16.8 (4.3)	20.3 (4.6)	26.9 (6.4)	<0.0001
SSF (right)	*n* = 197	*n* = 18	*n* = 82	*n* = 52	*n* = 45	
Mean (SD) (mm)	20.6 (7.7)	11.6 (2.4)	17.2 (5.1)	22.7 (5.7)	28.1 (7.5)	<0.0001
MAMC (right)	*n* = 198	*n* = 19	*n* = 82	*n* = 52	*n* = 45	
Mean (SD) (cm)	24.4 (2.9)	21.1 (1.4)	22.9 (1.6)	24.9 (1.5)	28.1 (2.2)	<0.0001
Wrist circumference (right)	*n* = 198	*n* = 19	*n* = 82	*n* = 52	*n* = 45	
Mean (SD) (cm)	15.8 (1.1)	14.7 (0.8)	15.4 (0.8)	15.9 (0.8)	16.9 (0.9)	<0.0001
Frame size	*n* = 198	*n* = 19	*n* = 82	*n* = 52	*n* = 45	
Mean (SD) (cm/cm)	10.1 (0.65)	10.5 (0.51)	10.3 (0.54)	10.0 (0.63)	9.6 (0.62)	<0.0001
Small frame (%)	10 (5.1)	3 (15.8)	5 (6.1)	2 (3.8)	0 (0.0)	0.0627
Medium frame (%)	92 (46.5)	11 (57.9)	52 (63.4)	22 (42.3)	7 (15.6)	<0.0001
Large frame (%)	96 (48.5)	5 (26.3)	25 (30.5)	28 (53.8)	38 (84.4)	<0.0001

Abbreviations: MAMC, mid arm muscle circumference; MUAC, mid upper arm circumference; SSF, subscapular skinfold; TSF, tricep skinfold.

^a^ BMI calculated by using maternal body weight post birth.

**TABLE 2:** Correlation between maternal age and anthropometric measurements.

Parameter	*r* Value	95% CI	*p*‐Value
BMI	0.2607	0.1266–0.3856	0.0002
MUAC (left)	0.2846	0.1515–0.4076	<0.0001
MUAC (right)	0.2775	0.1436–0.4014	<0.0001
TSF (right)	0.2377	0.1016–0.3651	0.0007
SSF (right)	0.2123	0.0747–0.3420	0.0027
MAMC (right)	0.2458	0.1101–0.3725	0.0005
WC (right)	0.1412	0.0018–0.2753	0.0472

### Accuracy of anthropometric measurements in identifying nutritional status

**FIGURE 1:**
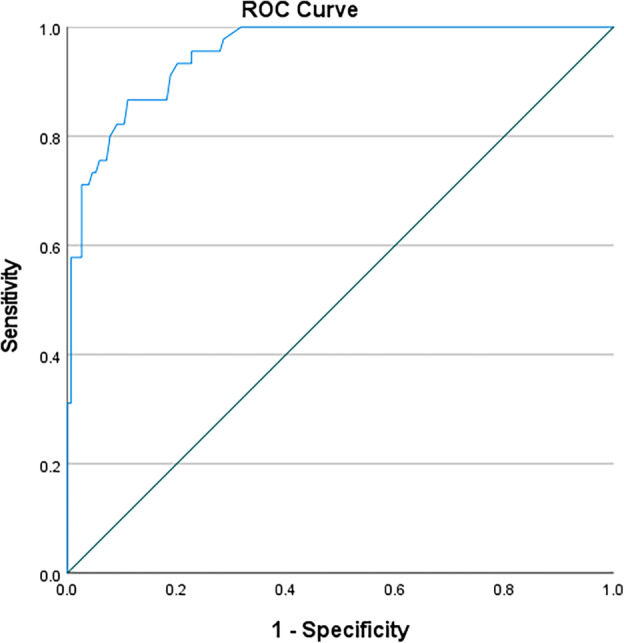
MUAC (left) accuracy in predicting obese nutritional status in pregnant black South African women using ROC curve.

**FIGURE 2:**
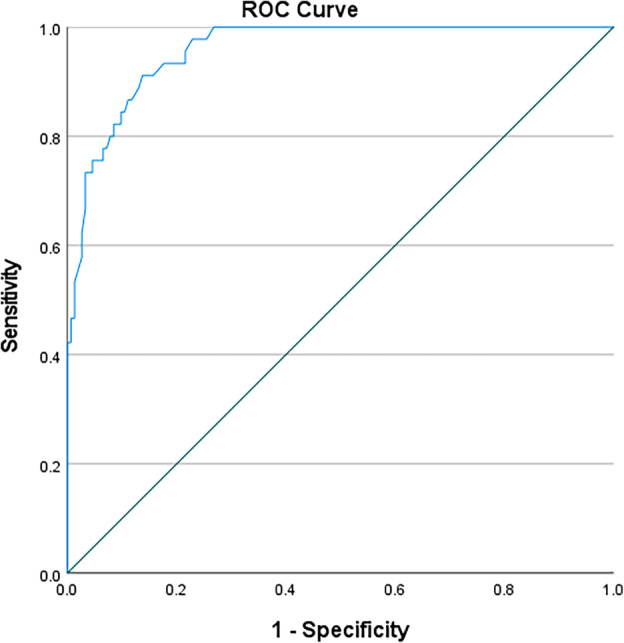
MUAC (right) accuracy in predicting obese nutritional status in pregnant black South African women using ROC curve.

**FIGURE 3:**
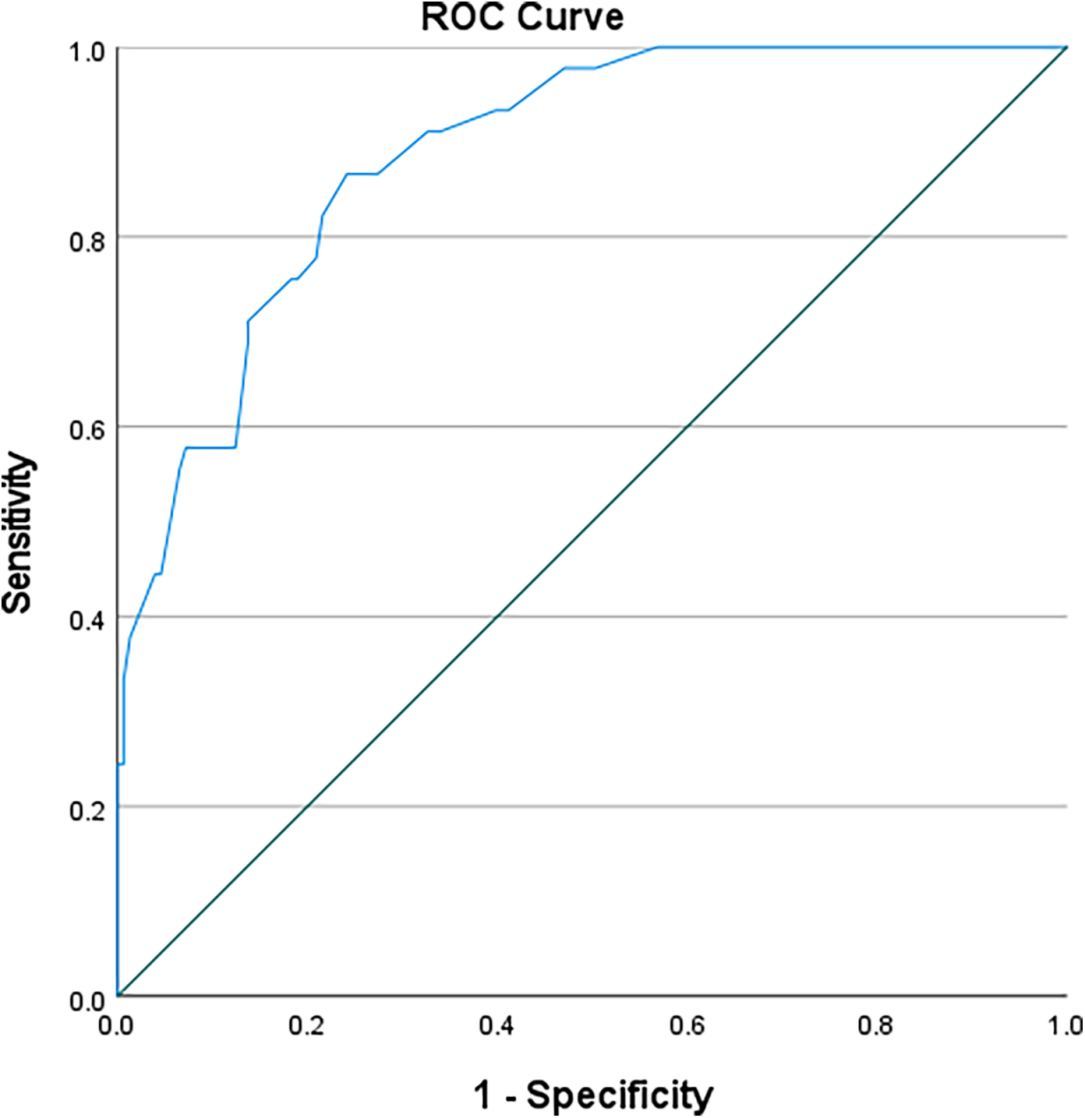
TSF (right) accuracy in predicting obese nutritional status in pregnant black South African women using ROC curve.

**FIGURE 4:**
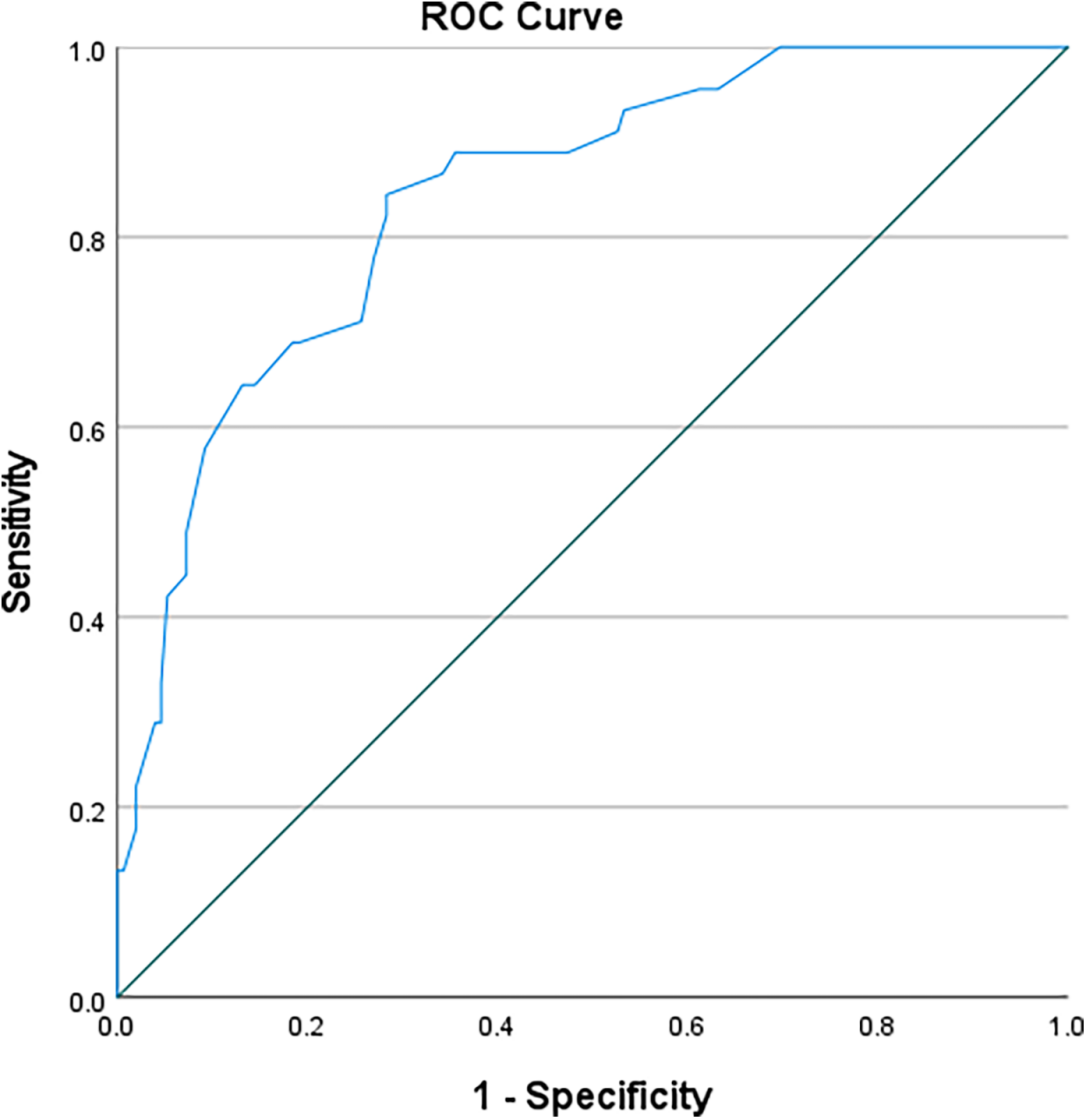
SSF (right) accuracy in predicting obese nutritional status in pregnant black South African women using ROC curve.

**FIGURE 5:**
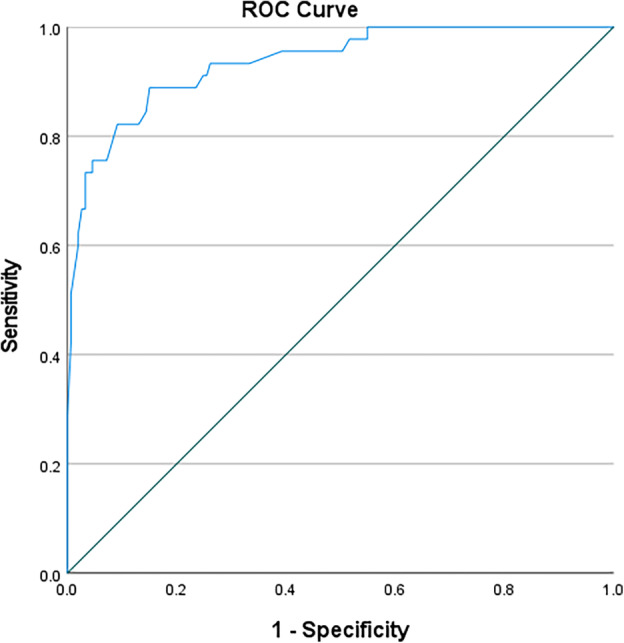
MAMC (right) accuracy in predicting obese nutritional status in pregnant black South African women using ROC curve.

In this study, ROC curves were used to test the accuracy of using anthropometric measurements for identifying the nutritional status of black South African pregnant women (Table 3). MUAC (left) was an outstanding predictive tool for obese nutritional status (AUC 0.952; 95%CI 0.924–0.980; *p* < 0.0001) with a cut‐off value of 32.30 cm (sensitivity 0.867, specificity 0.149, Youden's J statistic 0.016) (Figure 1). MUAC (right) was also an outstanding predictive tool for obese nutritional status (AUC 0.958; 95%CI 0.932–0.983; *p* < 0.0001) with a cut‐off value of 31.95 cm (sensitivity 0.933, specificity 0.176, Youden's J statistic 0.109) (Figure 2). TSF (right) was an excellent predictive tool for obese nutritional status (AUC 0.888; 95%CI 0.839–0.936; *p* < 0.0001) with a cut‐off value of 20.75 mm (sensitivity 0.867, specificity 0.242, Youden's J statistic 0.109) (Figure 3). SSF (right) was an excellent predictive tool for obese nutritional status (AUC 0.888; 95%CI 0.839–0.936; *p* < 0.0001) with a cut‐off value 21.75 mm (sensitivity 0.822, specificity 0.283, Youden's J statistic 0.105) (Figure 4). MAMC (right) was an outstanding predictive tool for obese nutritional status (AUC 0.935; 95%CI 0.895–0.976; *p* < 0.0001) with a cut‐off value of 25.23 mm (sensitivity 0.889, specificity 0.170, Youden's J statistic 0.059) (Figure 5). WC (right) was an excellent predictive tool for obese nutritional status (AUC 0.844; 95%CI 0.782–0.906; *p* < 0.0001) with a cut‐off value of 16.25 cm (sensitivity 0.711, specificity 0.131, Youden's J statistic −0.158). Frame size (cm/cm) was a poor predictive tool for all nutritional status groups.

**TABLE 3:** Summary of the accuracy of the anthropometric indicators for assessing nutritional status in pregnant black South African women.

Anthropometric indicator	Normal	Underweight	Overweight	Obese
MUAC (left) (cm)	X	X	≥28.55^a^	≥32.30^c^
MUAC (right) (cm)	X	X	≥28.75^a^	≥31.95^c^
TSF (right) (mm)	X	X	X	≥20.75^b^
SSF (right) (mm)	X	X	≥15.75^a^	≥21.75^b^
MAMC (right) (cm)	X	X	≥23.35^a^	≥25.23^c^
WC (right) (cm)	X	X	X	≥16.25^b^
Frame size (cm/cm)	≥9.95^a^	≥10.05^a^	X	X

*Note*: X, inaccurate, AUC <0.60.

Abbreviations: BMI: body mass index; MAMC, mid‐arm muscle circumference; MUAC, mid‐upper arm circumference; SSF, subscapular skinfold; TSF, tricep skinfold; WC, wrist circumference.

^a^ Poor accuracy, AUC ≥0.60 and <0.70.

^b^ Excellent accuracy, >0.80 and <0.90.

^c^ Outstanding accuracy, AUC ≥0.90.

### Anthropometric variation in pregnant women with and without HIV infection

The characteristics of the pregnant women were categorized according to HIV status (Table 4; Table S4). Of the 200 pregnant women in the study, 45.0% (*n* = 90) were HIV‐infected and 55.0% (*n* = 110) were HIV‐uninfected. The HIV‐infected pregnant women were older than the HIV‐uninfected pregnant women (*p* < 0.0001). Overall, there were no differences between the HIV‐infected pregnant women and the HIV‐uninfected pregnant women across all the anthropometric parameters.

**TABLE 4:** Anthropometric data for all pregnant females, categorized according to HIV status.

Characteristics	HIV‐infected		HIV‐uninfected	*p*‐Value
Age	*n* = 90		*n* = 110	<0.0001
Mean (SD) (years)	29.0 (5.5)		25.3 (5.1)	
Gestational age (SD)	*n* = 90		*n* = 110	0.0894
Mean (SD) (weeks)	37.4 (2.9)		37.9 (2.7)	
BMI Mean (SD) (kg/m^2^)	All pregnant women (*n* = 200)	*n* = 90	*n* = 110	0.6540
		31.7 (6.1)	32.5 (7.2)	
	Obese (*n* = 45)	*n* = 18	*n* = 27	0.8258
		40.4 (5.0)	42.0 (7.1)	
	Overweight (*n* = 52)	*n* = 24	*n* = 28	0.6529
		33.7 (1.9)	33.3 (1.6)	
	Normal (*n* = 84)	*n* = 38	*n* = 46	0.7226
		28.5 (2.1)	28.2 (2.4)	
	Underweight (*n* = 19)	*n* = 10	*n* = 9	0.7802
		22.9 (2.2)	23.3 (1.4)	
MUAC (left) Mean (SD) (cm)	All pregnant women (*n* = 200)	*n* = 90	*n* = 109	0.9605
		30.4 (4.6)	30.8 (5.5)	
	Obese (*n* = 45)	*n* = 18	*n* = 27	0.8894
		36.7 (3.9)	37.5 (5.7)	
	Overweight (*n* = 52)	*n* = 24	*n* = 28	0.5943
		31.4 (2.5)	31.3 (2.5)	
	Normal (*n* = 83)	*n* = 38	*n* = 45	0.2647
		28.3 (2.3)	27.7 (2.3)	
	Underweight (*n* = 19)	*n* = 10	*n* = 9	0.9024
		24.2 (1.5)	24.1 (1.8)	
MUAC (right) Mean (SD) (cm)	All pregnant women (*n* = 200)	*n* = 89	*n* = 109	0.7103
		30.4 (4.7)	31.0 (5.4)	
	Obese (*n* = 45)	*n* = 18	*n* = 27	0.7457
		36.9 (3.9)	37.8 (5.3)	
	Overweight (*n* = 52)	*n* = 24	*n* = 28	0.8328
		31.3 (2.1)	31.5 (2.5)	
	Normal (*n* = 82)	*n* = 37	*n* = 45	0.3810
		28.3 (2.4)	27.9 (2.4)	
	Underweight (*n* = 19)	*n* = 10	*n* = 9	0.6532
		24.2 (1.8)	24.6 (1.9)	
TSF (right) Mean (SD) (mm)	All pregnant women (*n* = 200)	*n* = 89	*n* = 109	0.5303
		18.9 (6.0)	19.9 (7.2)	
	Obese (*n* = 45)	*n* = 18	*n* = 27	0.2321
		25.1 (5.2)	28.0 (7.0)	
	Overweight (*n* = 52)	*n* = 24	*n* = 28	0.9707
		20.2 (4.6)	20.3 (4.7)	
	Normal (*n* = 82)	*n* = 37	*n* = 45	0.4865
		17.2 (4.5)	16.5 (4.2)	
	Underweight (*n* = 19)	*n* = 10	*n* = 9	0.5925
		10.8 (2.7)	11.5 (2.5)	
SSF (right) Mean (SD) (mm)	All pregnant women (*n* = 200)	*n* = 89	*n* = 108	0.5531
		20.4 (8.1)	20.8 (7.4)	
	Obese (*n* = 45)	*n* = 18	*n* = 27	0.7454
		27.9 (8.2)	28.3 (7.3)	
	Overweight (*n* = 52)	*n* = 24	*n* = 28	0.3772
		23.5 (6.2)	22.0 (5.4)	
	Normal (*n* = 82)	*n* = 37	*n* = 45	0.8923
		17.2 (5.8)	17.2 (4.6)	
	Underweight (*n* = 18)	*n* = 10	*n* = 8	0.5611
		11.1 (1.8)	12.3 (3.1)	
MAMC (right) Mean (SD) (cm)	All pregnant women (*n* = 200)	*n* = 89	*n* = 109	0.9781
		24.4 (2.8)	24.5 (2.9)	
	Obese (*n* = 45)	*n* = 18	*n* = 27	0.5463
		28.4 (1.8)	27.9 (2.5)	
	Overweight (*n* = 52)	*n* = 24	*n* = 28	
		25.0 (1.3)	25.0 (1.7)	0.9634
	Normal (*n* = 81)	*n* = 37	*n* = 44	0.4900
		23.0 (1.7)	22.8 (1.6)	
	Underweight (*n* = 19)	*n* = 10	*n* = 9	0.8377
		21.0 (1.6)	21.2 (1.3)	
Wrist circumference (right) Mean (SD) (cm)	All pregnant women (*n* = 200)	*n* = 89	*n* = 109	0.5361
		15.7 (1.0)	15.9 (1.2)	
	Obese (*n* = 45)	*n* = 18	*n* = 27	0.9260
		16.7 (0.7)	17.0 (1.3)	
	Overweight (*n* = 52)	*n* = 24	*n* = 28	0.2900
		15.8 (0.8)	16.0 (0.9)	
	Normal (*n* = 82)	*n* = 37	*n* = 45	0.9368
		15.4 (1.0)	15.4 (0.7)	
	Underweight (*n* = 19)	*n* = 10	*n* = 9	0.9674
		14.8 (0.9)	14.7 (0.8)	
**Frame size**	All pregnant women (*n* = 200)	*n* = 89	*n* = 109	0.6128
Mean (SD) (cm)		10.13 (0.58)	10.08 (0.71)	
Small frame		3 (3.4)	7 (6.4)	0.5165
Medium frame		44 (49.4)	48 (44.0)	0.4762
Large frame		42 (47.2)	54 (49.5)	0.7762

Abbreviations: BMI, body mass index; MAMU, mid arm muscle circumference; MUAC, mid upper arm circumference; SSF, subscapular skinfold; TSF, Tricep skinfold.

## DISCUSSION

It has been well documented that women gain weight during pregnancy, especially fat mass, with obese pregnant women having the largest fat stores compared to other nutritional statuses.^36^, ^37^, ^38^, ^39^ This study supported this by showing that an increase in maternal BMI was correlated with an increase in the size of other maternal anthropometric measurements, with MUAC (left and right) being the most accurate predictive tool for BMI. This is similar to the findings of other African studies, which identified that MUAC could be used as a potential nutritional status indicator in obese pregnant and malnourished pregnant women.^10^, ^14^, ^40^ To date, our study is the first African study to have used skinfolds measurements, MAMC or WC to assess nutritional status in pregnant women. These measurements are shown to be reliable measurements for indicators body fat percentage in pregnant women^13^ and were correlated with BMI in our study population.

Our findings identified that a rise in maternal age was associated with an increase in BMI as well as an associated increase in all other anthropometric measurements including MUAC (left and right), TSF (right), SSF (right), MAMC (right), and WC (right). It is well known that advanced maternal age has been linked to an increased risk for adverse pregnancy outcomes.^40^ A possible contributing factor for this risk associated with advance in maternal age may be due to the associated reduction in the resting metabolic rate which in turn leads to body composition changes, such as an increase in fat stores and decrease in muscle stores.^41^

Despite the large numbers of women of reproductive age in Sub‐Saharan Africa who live with HIV, few studies have investigated the relationship between HIV infection during pregnancy and maternal anthropometric parameters of nutritional status. In this study, the HIV‐infected pregnant women consistently had lower measurements in comparison to the HIV‐uninfected pregnant women, however, there were no significant differences between any of the anthropometric measurements. Overall, HIV‐infected pregnant women did not differ anthropometrically from their HIV‐uninfected counterparts. One of the potential reasons for these results could be due to the ART compliance of the study population, which then prevents HIV replication and prevention of the associated changes in nutritional status such as in HIV‐associated wasting syndrome. Wasting syndrome is defined as a complication of HIV infection, where a specific combination of internal factors leads to a hypermetabolic response and initiates a catabolic effect, breaking down muscle and fat tissue.^42^ The internal factors that mediate this process will vary from patient to patient but may include compliance to the ART program, dietary patterns, malabsorption, physical activity, metabolic derangements, epigenetics, and cytokine activity.^42^

This study is a population‐specific study, allowing the study findings to give a novel insight into the exposures and outcomes associated with overweight and obesity in pregnant black South African women. However, this study was limited by not including pre‐pregnancy maternal weight as well as not using bioelectrical impedance to calculate body fat percentage.

In conclusion, surrogate anthropometric measurements offer a simple solution for assessing nutritional status in pregnant women. In this population‐specific study, MUAC was the most accurate method for identifying overweight and obesity. Furthermore, pregnant women living with HIV do not differ anthropometrically to pregnant women living without HIV.

## AUTHOR CONTRIBUTIONS

**Christen R. Erasmus**, **Niren R. Maharaj**, and **Anil A. Chuturgoon** conceived and designed the study. **Christen R. Erasmus** conducted all the data collection, analysis, and writing of the first draft of the paper. **Christen R. Erasmus**, **Niren R. Maharaj**, and **Anil A. Chuturgoon** contributed to the editing of the paper. All authors critically reviewed the manuscript and approved the final version submitted for publication.

## CONFLICT OF INTEREST STATEMENT

The authors declare there was no personal or commercial conflict of interest in the performance of the present study.

## Supporting information


**Table S1:** Summary of the interpretation of percentile readings for MUAC, TSF, SSF, and MAMC.

**Table S2:** Interpretation of frame size for females.

**Table S3:** Anthropometric percentile readings for pregnant black South African females, categorized according to nutritional status.

**Table S4:** Anthropometric percentile readings for all pregnant females, categorized according to HIV status.

## Data Availability

The data that support the findings of this study are available on request from the corresponding author. The ethical approval granted for this study by the research ethics committee stipulates that the raw data must be kept confidential to protect participant privacy.
