# Supplementary material for: Anthropometric measurements as predictors of nutritional status in black South African women during pregnancy
Source: J Obstet Gynaecol Res. 2024 Dec 25;51(1):e16184. doi: 10.1111/jog.16184 (PMC11669476; doi:10.1111/jog.16184)
Supplement: Supplementary file 1 — Table S1: Summary of the interpretation of percentile readings for MUAC, TSF, SSF, and MAMC. [file JOG-51-0-s001.docx]

1. *Supplementary Table 1: Summary of the interpretation of percentile readings for MUAC, TSF, SSF, and MAMC*

| Percentile reading: | MUAC | TSF | SSF | MAMC |
| --- | --- | --- | --- | --- |
| ≤ 5^th^ | Very thin arm size | Very low-fat stores | Very low-fat stores | Very low muscle stores |
| ≤ 10^th^ | Very thin arm size | Very low-fat stores | Very low-fat stores | Very low muscle stores |
| ≤ 25^th^ | Thin arm size | Low-fat stores | Low-fat stores | Low muscle stores |
| ≤ 50^th^ | Normal arm size | Low-fat stores | Low-fat stores | Low muscle stores |
| ≤ 75^th^ | Thick arm size | High-fat stores | High-fat stores | High muscle stores |
| ≤ 90^th^ | Thick arm size | High-fat stores | High-fat stores | High muscle stores |
| ≤ 95^th^ | Very thick arm size | Very High-fat stores | Very High-fat stores | Very High muscle stores |
| ≥ 95^th^ | Very thick arm size | Very High-fat stores | Very High-fat stores | Very High muscle stores |
| *Abbreviations: MUAC: Mid-upper arm circumference; TSF: Tricep skinfold; SSF: Subscapular skinfold; MAMC: Mid-arm muscle circumference* | | | | |
