# Supplementary material for: Anthropometric measurements as predictors of nutritional status in black South African women during pregnancy
Source: J Obstet Gynaecol Res. 2024 Dec 25;51(1):e16184. doi: 10.1111/jog.16184 (PMC11669476; doi:10.1111/jog.16184)
Supplement: Supplementary file 2 — Table S2: Interpretation of frame size for females. [file JOG-51-0-s002.docx]

*Supplementary Table 2: Interpretation of frame size for females*

| Classification: | Calculated measurement (cm/cm) in females |
| --- | --- |
| Small body frame | >11.0 |
| Medium body frame | 10.1 – 11.0 |
| Large body frame | <10.1 |
