# Supplementary material for: Anthropometric measurements as predictors of nutritional status in black South African women during pregnancy
Source: J Obstet Gynaecol Res. 2024 Dec 25;51(1):e16184. doi: 10.1111/jog.16184 (PMC11669476; doi:10.1111/jog.16184)
Supplement: Supplementary file 3 — Table S3: Anthropometric percentile readings for pregnant black South African females, categorized according to nutritional status. [file JOG-51-0-s003.docx]

*Supplementary Table 3: Anthropometric percentile readings for pregnant black South African females, categorized according to nutritional status*

| Characteristics | All pregnant females | Maternal BMI^a^ | | | | |
| --- | --- | --- | --- | --- | --- | --- |
|  |  | Underweight | Normal | Overweight | Obese | p-value |
| MUAC^b^ (right) | n=198 | n=19 | n=82 | n=52 | n=45 |  |
| <5^th^ (very thin) (%) | 4 (2.0) | 4 (21.1) | 0 (0.0) | 0 (0.0) | 0 (0.0) | <0.0001 |
| < 10^th^ (very thin) (%) | 1 (0.5) | 1 (0.1) | 0 (0.0) | 0 (0.0) | 0 (0.0) | 0.0237 |
| < 25^th^ (thin) (%) | 11 (5.6) | 6 (31.6) | 5 (6.1) | 0 (0.0) | 0 (0.0) | <0.0001 |
| < 50^th^ (normal) (%) | 39 (23.2) | 7 (36.8) | 30 (36.6) | 2 (3.8) | 0 (0.0) | <0.0001 |
| <75^th^ (thick) (%) | 39 (23.2) | 1 (0.1) | 28 (34.1) | 10 (19.2) | 0 (0.0) | <0.0001 |
| < 90^th^ (thick) (%) | 59 (29.8) | 0 (0.0) | 17 (20.7) | 31 (59.6) | 11 (24.4) | <0.0001 |
| < 95^th^ (very thick) (%) | 20 (10.1) | 0 (0.0) | 1 (1.2) | 8 (15.4) | 11 (24.4) | 0.0001 |
| >95^th^ (very thick) (%) | 25 (12.6) | 0 (0.0) | 1 (1.2) | 1 (1.9) | 23 (51.1) | <0.0001 |
| TSF^c^ (right) | n=198 | n=19 | n=82 | n=52 | n=45 |  |
| <5^th^ (very low) (%) | 10 (5.1) | 8 (42.1) | 2 (2.4) | 0 (0.0) | 0 (0.0) | <0.0001 |
| < 10^th^ (very low) (%) | 20 (10.1) | 5 (26.3) | 14 (17.1) | 1 (1.9) | 0 (0.0) | 0.0003 |
| < 25^th^ (low) (%) | 60 (30.3) | 5 (26.3) | 32 (39.0) | 20 (38.5) | 3 (6.7) | 0.0008 |
| < 50^th^ (normal) (%) | 62 (31.3) | 1 (5.3) | 28 (34.1) | 16 (30.8) | 17 (37.8) | 0.0663 |
| <75^th^ (high) (%) | 36 (18.2) | 0 (0.0) | 6 (7.3) | 15 (28.8) | 15 (33.3) | <0.0001 |
| < 90^th^ (high) (%) | 4 (2.0) | 0 (0.0) | 0 (0.0) | 0 (0.0) | 4 (8.9) | 0.0031 |
| < 95^th^ (very high) (%) | 3 (1.5) | 0 (0.0) | 0 (0.0) | 0 (0.0) | 3 (6.7) | 0.0158 |
| >95^th^ (very high) (%) | 3 (1.5) | 0 (0.0) | 0 (0.0) | 0 (0.0) | 3 (6.7) | 0.0158 |
| SSF^d^ (right) | n=197 | n=18 | n=82 | n=52 | n=45 |  |
| <5th (very low) (%) | 13 (6.6) | 7 (38.9) | 5 (6.1) | 1 (1.9) | 0 (0.0) | <0.0001 |
| < 10th (very low) (%) | 10 (5.1) | 5 (27.8) | 4 (4.9) | 0 (0.0) | 1 (2.2) | <0.0001 |
| < 25th (low) (%) | 84 (42.6) | 5 (27.8) | 48 (58.5) | 23 (44.2) | 8 (17.8) | <0.0001 |
| < 50th (normal) (%) | 53 (26.9) | 0 (0.0) | 18 (22.0) | 21 (40.4) | 14 (31.1) | 0.0050 |
| <75th (high) (%) | 10 (5.1) | 1 (5.6) | 5 (6.1) | 2 (3.8) | 2 (4.4) | 0.9430 |
| < 90th (high) (%) | 15 (7.6) | 0 (0.0) | 1 (1.2) | 3 (5.8) | 11 (24.4) | <0.0001 |
| < 95th (very high) (%) | 0 (0.0) | 0 (0.0) | 0 (0.0) | 0 (0.0) | 0 (0.0) | - |
| >95th (very high) (%) | 12 (6.1) | 0 (0.0) | 1 (1.2) | 2 (3.8) | 9 (20.0) | 0.0009 |
| MAMC^e^ (right) | n=198 | n=19 | n=82 | n=52 | n=45 |  |
| <5th (very low) (%) | 0 (0.0) | 0 (0.0) | 0 (0.0) | 0 (0.0) | 0 (0.0) | - |
| < 10th (very low) (%) | 0 (0.0) | 0 (0.0) | 0 (0.0) | 0 (0.0) | 0 (0.0) | - |
| < 25th (low) (%) | 4 (2.0) | 3 (15.8) | 0 (0.0) | 0 (0.0) | 1 (2.2) | <0.0001 |
| < 50th (normal) (%) | 18 (9.1) | 5 (26.3) | 11 (13.4) | 2 (3.8) | 0 (0.0) | 0.0019 |
| <75th (high) (%) | 39 (19.7) | 8 (42.1) | 29 (35.4) | 2 (3.8) | 0 (0.0) | <0.0001 |
| < 90th (high) (%) | 53 (26.8) | 3 (15.8) | 28 (34.1) | 19 (36.5) | 3 (6.6) | 0.0016 |
| < 95th (very high) (%) | 30 (15.2) | 0 (0.0) | 10 (12.2) | 15 (28.8) | 5 (11.1) | 0.0070 |
| >95th (very high) (%) | 54 (27.3) | 0 (0.0) | 4 (4.9) | 14 (26.9) | 36 (80.0) | <0.0001 |
| *Notes: ^a^BMI calculated by using maternal body weight post birth; ^b^Mid upper arm circumference; ^c^Tricep skinfold; ^d^Subscapular skinfold; ^e^Mid arm muscle circumference* | | | | | | |
