# Supplementary material for: Anthropometric measurements as predictors of nutritional status in black South African women during pregnancy
Source: J Obstet Gynaecol Res. 2024 Dec 25;51(1):e16184. doi: 10.1111/jog.16184 (PMC11669476; doi:10.1111/jog.16184)
Supplement: Supplementary file 4 — Table S4: Anthropometric percentile readings for all pregnant females, categorized according to HIV status. [file JOG-51-0-s004.docx]

Supplementary Table 4: Anthropometric percentile readings for all pregnant females, categorized according to HIV status

| **Characteristics** | **HIV-infected** | | **HIV-uninfected** | **p-value** |
| --- | --- | --- | --- | --- |
| **MUAC^b^ (right)**  Mean (SD) (cm) | **All pregnant women (n=200)** | **n=89** | **n=109** |  |
|  | <5th (very thin) (%) | 3 (3.4) | 1 (0.9) | 0.3281 |
|  | < 10th (very thin) (%) | 1 (1.1) | 0 (0.0) | 0.4495 |
|  | < 25th (thin) (%) | 8 (9.0) | 3 (2.8) | 0.0674 |
|  | < 50th (normal) (%) | 16 (18.0) | 23 (21.1) | 0.5962 |
|  | <75th (thick) (%) | 15 (16.9) | 24 (22.0) | 0.3767 |
|  | < 90th (thick) (%) | 30 (33.7) | 29 (26.6) | 0.3488 |
|  | < 95th (very thick) (%) | 8 (9.0) | 12 (11.0) | 0.8133 |
|  | >95th (very thick) (%) | 8 (9.0) | 17 (15.6) | 0.2819 |
| **TSF^c^ (right)**  Mean (SD) (mm) | **All pregnant women (n=200)** | **n=89** | **n=109** |  |
|  | <5^th^ (very low) (%) | 6 (6.7) | 4 (3.7) | 0.3495 |
|  | < 10^th^ (very low) (%) | 9 (10.1) | 11 (10.1) | 1.000 |
|  | < 25^th^ (low) (%) | 25 (28.1) | 35 (32.1) | 0.6412 |
|  | < 50^th^ (normal) (%) | 32 (36.0) | 30 (27.5) | 0.2205 |
|  | <75^th^ (high) (%) | 14 (15.7) | 22 (20.2) | 0.4628 |
|  | < 90^th^ (high) (%) | 2 (2.2) | 2 (1.8) | 1.0000 |
|  | < 95^th^ (very high) (%) | 1 (1.1) | 2 (1.8) | 1.0000 |
|  | >95^th^ (very high) (%) | 0 (0.0) | 3 (2.8) | 0.2539 |
| **SSF^d^ (right)**  Mean (SD) (mm) | **All pregnant women (n=200)** | **n=89** | **n=108** |  |
|  | <5th (very low) (%) | 9 (10.1) | 4 (3.7) | 0.0874 |
|  | < 10th (very low) (%) | 6 (6.7) | 4 (3.7) | 0.3519 |
|  | < 25th (low) (%) | 33 (37.1) | 51 (47.2) | 0.1926 |
|  | < 50th (normal) (%) | 27 (30.3) | 26 (24.1) | 0.3374 |
|  | <75th (high) (%) | 3 (3.4) | 7 (6.5) | 0.5165 |
|  | < 90th (high) (%) | 6 (6.7) | 9 (8.3) | 0.7903 |
|  | < 95th (very high) (%) | 0 (0.0) | 0 (0.0) | - |
|  | >95^th^ (very high) % | 5 (5.6) | 7 (6.5) | 1.000 |
| **MAMC^e^ (right)**  Mean (SD) (cm) | **All pregnant women (n=200)** | **n=89** | **n=109** |  |
|  | <5th (very low) (%) | 0 (0.0) | 0 (0.0) | - |
|  | < 10th (very low) (%) | 0 (0.0) | 0 (0.0) | - |
|  | < 25th (low) (%) | 3 (3.4) | 1 (0.9) | 0.3281 |
|  | < 50th (normal) (%) | 12 (13.5) | 6 (5.5) | 0.0798 |
|  | <75th (high) (%) | 13 (14.6) | 26 (23.9) | 0.1103 |
|  | < 90th (high) (%) | 24 (27.0) | 29 (26.6) | 1.0000 |
|  | < 95th (very high) (%) | 16 (18.0) | 14 (12.8) | 0.3273 |
|  | >95^th^ (very high) % | 21 (23.6) | 33 (30.3) | 0.3374 |
| *^a^Body mass index; ^b^Mid upper arm circumference; ^c^Tricep skinfold; ^d^Subscapular skinfold; ^e^Mid arm muscle circumference* | | | | |
